# Editorial: Mobilization of hematopoietic cells from the bone marrow to the peripheral blood: Challenges and new therapeutic targets

**DOI:** 10.3389/fphar.2025.1592559

**Published:** 2025-04-22

**Authors:** Agata Szade, Gunsagar S. Gulati, Agnieszka D. Czechowicz

**Affiliations:** ^1^ Department of Medical Biotechnology, Faculty of Biochemistry, Biophysics and Biotechnology, Jagiellonian University, Krakow, Poland; ^2^ Department of Medical Oncology Dana-Farber Cancer Institute, Boston, MA, United States; ^3^ Division of Hematology, Oncology, Stem Cell Transplantation and Regenerative Medicine, Department of Pediatrics, Stanford University School of Medicine, Stanford, CA, United States; ^4^ Institute for Stem Cell Biology and Regenerative Medicine, Stanford University School of Medicine, Stanford, CA, United States; ^5^ Center for Definitive and Curative Medicine, Stanford University School of Medicine, Stanford, CA, United States

**Keywords:** hematopoietic stem and progenitor cells (HPSCs), mobilization of hematopoietic cells, graft vs host disease, conditioning regimens for HCT, hematopoietic cell transplantation (HCT)

Every year, hematopoietic cell transplantation (HCT) saves the lives of tens of thousands of patients world-wide. HCT is primarily used to treat hematologic malignancies and non-malignant bone marrow disorders, as well as blood- and immune-related genetic conditions such as hemoglobinopathies and severe combined immunodeficiency (Atsuta et al., 2024). It is also occasionally used in the treatment of certain solid tumors, such as neuroblastoma (Storb et al., 2003). Advances in gene therapy and gene-editing technologies are further expanding the potential applications of HCT (Haltalli et al., 2022). However, there remains a pressing need to refine transplant procedures and improve their safety for recipients.

In the early days of HCT, bone marrow aspirates were the sole source of hematopoietic stem cells (HSCs) for transplantation. While bone marrow harvests remain the preferred graft source for certain uses, such as pediatric transplants and non-malignant disorders, mobilized peripheral blood has become the primary graft source for approximately 90% of transplants (Atsuta et al., 2024; Arai and Klingemann, 2003; Passweg et al., 2021). In this approach, donors receive mobilizing factors that stimulate the release of HSCs from the bone marrow into the bloodstream, making collection safer and more convenient. Umbilical cord blood (UCB)-derived hematopoietic stem and progenitor cells (HSPCs) are also used as an alternative graft source for HCT (Atsuta et al., 2024; Passweg et al., 2021). However, a key challenge with UCB—and sometimes bone marrow and mobilized blood—is obtaining a sufficient number of HSPCs (To et al., 2011; Kuang et al., 2022; Ahn et al., 2024). Research is ongoing to develop methods to increase the number of mobilized cells in donors (Luo et al., 2021; Hoggatt et al., 2018; Szade et al., 2019), reliably expand HSPCs *ex vivo*, and improve HSPC survival during and after transplantation.

Two studies in this collection address these challenges. Li et al. describe the *ex vivo* expansion of UCB-derived HSCs using the small molecule chrysin, which delays HSC differentiation and inhibits apoptosis induced by reactive oxygen species (ROS). Prchal-Murphy et al. report a strategy that combines existing drugs to enhance the survival of umbilical cord-derived HSPCs. Their work demonstrates that sequential priming of murine or human HSPCs with treprostinil and forskolin, followed by cinacalcet treatment in recipients, accelerates hematopoietic recovery and improves survival in transplanted mice.

From the recipient’s perspective, effective conditioning is crucial for replacement of the hematolymphoid system and engraftment of transplanted cells. Conditioning removes the patient’s endogenous HSCs from their bone marrow niches, eliminating the recipient’s diseased cells and creating space for the transplanted graft (Szade et al., 2018). In cancer patients undergoing HCT, conditioning is also needed to deplete residual cancer cells and minimize the risk of recurrence before transplanting isolated cells. This is typically achieved using chemotherapy, total body irradiation (TBI), or a combination of both. The choice of regimen depends on factors such as graft used, the type of disease, remission status, and patient-specific factors, such as age, comorbidities, organ function, prior treatments, and the risk of transplant-related complications (Gyurkocza and Sandmaier, 2014). Busulfan combined with cyclophosphamide is a commonly used myeloablative conditioning regimen in allogeneic HCT. (Gyurkocza and Sandmaier, 2014; Sugita and Yanada, 2024). However, Shi et al. report on a Phase I study evaluating the safety and efficacy of various myeloablative conditioning regimens, including busulfan, cyclophosphamide, cytarabine, and a purine nucleoside analog, cladribine, for autologous HCT in acute myeloid leukemia.

Non-genotoxic therapies are being explored for transplant conditioning, such as antibodies that block interactions between HSCs and their niches (Czechowicz et al., 2007; Griffin et al., 2022). In cancer patients, however, conditioning also serves to eliminate tumor cells though studies have shown that the donor immune response against residual tumor cells—known also as graft-versus-tumor (GVT) or graft-versus-leukemia (GVL)—is critical in reducing recurrence risk (Gyurkocza and Sandmaier, 2014; Sugita and Yanada, 2024). Unfortunately, the immune reaction of donor cells against the recipient’s tissues, termed graft-versus-host disease (GVHD), remains a serious complication of allogeneic HCT and occurs more frequently with mobilized peripheral blood grafts (Atsuta et al., 2024; Nassereddine et al., 2017). Clinical data show that the risk of chronic GVHD is higher when transplanting mobilized blood compared to bone marrow (Flowers and Martin, 2015; Zhang et al., 2023; Lacan et al., 2024). To prevent and treat GVHD, various immunosuppressive drugs, primarily corticosteroids, are used. However, many patients develop steroid-refractory GVHD, which is challenging to treat (Flinn and Gennery, 2023). New drugs are being investigated and introduced for this condition (Martini et al., 2022). Among these, ruxolitinib—a JAK inhibitor originally approved for treatment of myelofibrosis—has shown promise. In this Research Topic, Zhang et al. explore its effectiveness in treating bronchiolitis obliterans syndrome (BOS), a pulmonary manifestation of GVHD. Another study by Liu et al. focuses on optimizing dosing and pharmacokinetics of tacrolimus, a calcineurin inhibitor, in pediatric HCT recipients.

Hematopoietic cell transplantation is a curative treatment for many blood and immune diseases, though treatment approaches are constantly evolving. Especially as mobilized peripheral blood becomes the primary source of transplanted HSCs, it is crucial to enhance our understanding of GVHD mechanisms, the interplay between GVHD and GVT, and effective strategies for obtaining high-quality, transplantable HSCs. While many questions remain unanswered, we hope this Research Topic will offer valuable new insights.
